# Overexpression of *Arabidopsis AnnAt8* Alleviates Abiotic Stress in Transgenic *Arabidopsis* and Tobacco

**DOI:** 10.3390/plants5020018

**Published:** 2016-04-14

**Authors:** Deepanker Yadav, Israr Ahmed, Pawan Shukla, Prasanna Boyidi, Pulugurtha Bharadwaja Kirti

**Affiliations:** Department of Plant Sciences, School of Life Sciences, University of Hyderabad, Hyderabad 500 046, India; iahmed67@gmail.com (I.A.); shklpwn@gmail.com (P.S.); prasanna89boyidi@gmail.com (P.B.)

**Keywords:** annexin, seedling growth, osmotic stress, methyl viologen stress, salt stress, seed germination

## Abstract

Abiotic stress results in massive loss of crop productivity throughout the world. Because of our limited knowledge of the plant defense mechanisms, it is very difficult to exploit the plant genetic resources for manipulation of traits that could benefit multiple stress tolerance in plants. To achieve this, we need a deeper understanding of the plant gene regulatory mechanisms involved in stress responses. Understanding the roles of different members of plant gene families involved in different stress responses, would be a step in this direction. *Arabidopsis*, which served as a model system for the plant research, is also the most suitable system for the functional characterization of plant gene families. Annexin family in *Arabidopsis* also is one gene family which has not been fully explored. Eight annexin genes have been reported in the genome of *Arabidopsis thaliana*. Expression studies of different *Arabidopsis* annexins revealed their differential regulation under various abiotic stress conditions. *AnnAt8* (At5g12380), a member of this family has been shown to exhibit ~433 and ~175 fold increase in transcript levels under NaCl and dehydration stress respectively. To characterize Annexin8 (AnnAt8) further, we have generated transgenic *Arabidopsis* and tobacco plants constitutively expressing AnnAt8, which were evaluated under different abiotic stress conditions. *AnnAt8* overexpressing transgenic plants exhibited higher seed germination rates, better plant growth, and higher chlorophyll retention when compared to wild type plants under abiotic stress treatments. Under stress conditions transgenic plants showed comparatively higher levels of proline and lower levels of malondialdehyde compared to the wild-type plants. Real-Time PCR analyses revealed that the expression of several stress-regulated genes was altered in AnnAt8 over-expressing transgenic tobacco plants, and the enhanced tolerance exhibited by the transgenic plants can be correlated with altered expressions of these stress-regulated genes. Our findings suggest a role for AnnAt8 in enhancing abiotic stress tolerance at different stages of plant growth and development.

## 1. Introduction

Abiotic stress includes physical and chemical changes in the environment, which affect normal growth and productivity of the plant. Due to shifts in the climatic conditions and improper management in farming, abiotic stress has become a challenge for the global agriculture industry. Abiotic stress ultimately could lead to the loss of cell homeostasis and cell death. In order to maintain stability in cell structure and functional machinery inside the cell [1], plants have evolved a group of genes, whose expression helps them survive the adverse conditions [2]. Salinity and drought are common stresses, which plants face during their life cycle. Few plant species have evolved in such a way that they have developed adaptations, and can complete their life cycle under these stressful conditions. However, in other species, their defense system becomes operational when exposed to the stress [3,4,5]. The defense responses also vary with the severity and duration of stress given to a plant. In mild stress conditions, expressions of stress-induced genes do not overlap each other. However, under severe stress conditions, a common set of genes may upregulate, which could be due to the generation of reactive oxygen species (ROS) [6]. Many genes involved in defense mechanisms against abiotic stresses have been identified so far. Annexins are also among them. They are present throughout plants, animals and fungi. Recently their presence has also been reported in prokaryotes. Annexin is a superfamily of genes, and plant annexins which belong to family D, have been further grouped into different subfamilies [7,8]. In the course of evolution, plant annexins have become diverse in their structure and function [8]. They have been shown to play a significant role in plant growth and development under normal and stress conditions [8,9,10,11]. In plants, the first multigene annexin family was identified in *Arabidopsis* with eight members representing this family, and expression and characterization studies have established them as a multifunctional protein family [8,11,12,13,14,15,16,17,18]. They possess different properties like Ca^2+^ binding, ion permeability, and peroxidase activity, which have been correlated with their responses during plant development and stress condition [19]. An increase in plant annexin abundance and their recruitment to membrane has been reported under different stress conditions [20,21,22,23]. This recruitment of annexins to the membrane has been linked to many functions like channel properties, protection of membrane and ROS-induced signaling [24,25]. Another important property of annexin is Ca^2+^ dependent lipid binding. Recent reports suggest that annexins can mediate the ROS-induced changes in Ca^2+^ permeability of membrane [26,27,28,29]. This annexin-mediated Ca^2+^_(Cyt)_ modulation is supposed to play a significant role in abiotic stress signaling and gene regulation. Annexin regulatory responses can also be mediated by changes in phytohormone level during plant growth and development in stress conditions [14]. There is limited information on how these properties work in a cumulative manner during stress responses. Previous expression studies of annexin family members in different plants showed differential expression pattern under normal and stress conditions [12,16,30,31,32,33,34,35,36,37]. Proteomic studies in many plant species also displayed their upregulation under salinity [18,38,39] and heat stress [15,40,41]. In a transcriptome study in *Arabidopsis*, annexin members showed a differential expression pattern during seed and seedling stages, and most of them were shown to be up-regulated in various abiotic stress treatments [31]. Previous studies on AnnAt1, AnnAt2, and AnnAt4 have indicated their role in salt and drought tolerance [14,20,21], whereas AnnAt5 plays a role in pollen development [16,17]. Among the other members of *Arabidopsis* annexin family, *AnnAt8* (AT5G12380) has been reported for its high fold transcript accumulation during seedling stage under salt and dehydration stress and showed similar response comparable to *Rd29* and *P5Cs* (two marker genes for salt stress in *Arabidopsis*) [31]. Some tissue-specific gene expression studies in *Arabidopsis* also showed the presence of *AnnAt8* transcript in female gametophyte [42,43]. In a recent study, it has been demonstrated that *AnnAt8* expression in *Arabidopsis* roots does not change on treatment with H_2_O_2_ [29]. Above mentioned expression studies suggest a possible role of AnnAt8 in plant growth and development in normal as well as in stress conditions. Since gene expression studies do not always lead to conclusions about the gene function, we followed the overexpression strategy of gene characterization. Further to this, we generated *AnnAt8* overexpressing transgenic tobacco and *Arabidopsis* plants and analyzed their performance under different stress conditions. The current study provides evidence for the involvement of AnnAt8 in alleviating abiotic stress in transgenic *Arabidopsis* and tobacco.

## 2. Materials and Methods

### 2.1. AnnAt8 Construct Preparation

The full-length cDNA of the *AnnAt8* gene (Locus: At5g12380 and NCBI GenBank Accession No: NM_121276) was amplified by using following primers (ORF1 For and ORF1 Rev harboring *Nco*I and *Xba*I sites respectively) and cloned in plant expression vector pRT100. Primers employed for the amplification of ORFI are listed in Supplementary Table S1. Expression cassette with CaMV35S promoter and poly-adenylation signal was released from the pRT100 using *Pst*I digestion, and the same site was used to clone the expression cassette into the binary vector pCAMBIA2300.

### 2.2. Plant Material and Experimental Conditions

*Arabidopsis thaliana* ecotype Col 0 was used for generating the transgenic plants constitutively expressing AnnAt8. The *Arabidopsis* plants were grown in a growth room under standard controlled light conditions. For vegetative growth of plants controlled temperature (21 ± 2 °C) and light conditions (8 h of light/16 h of dark period) were used in the growth chamber. For reproductive growth, 16 h of light/8 h of dark period was used. Light intensity in growth chamber was maintained at 100–150 µmol·m^−2^·s^−1^.

*Nicotiana tabacum* Linn cultivar Samsun was used for transgenic generation. The tobacco plants were grown in the greenhouse under natural light conditions. Seed germination was performed, and seedlings were maintained in a growth room at 26 ± 2 °C with 16 h of light/8 h of dark.

### 2.3. Plant Transformation

*Arabidopsis* plant transformation was performed by the floral dip method with minor modifications [44]. *Agrobacterium* strain EHA 105 harboring the construct pCAMBIA2300::AnnAt8 was used to transform the *Arabidopsis* following standard *in planta* protocol [44]. For the floral dip transformation, an appropriate flowering stage was selected, and a bunch of flowering stalks was inverted to dip in *Agrobacterium* cell (harboring pCAMBIA2300::AnnAt8) suspension containing 5% sucrose and 0.05% Silwet L-77 for two min. Further, the plants were wrapped with plastic films to maintain high humidity for 16–24 h in the dark. Finally, the plastic covers were removed, and plants were allowed to grow to silique formation and maturity, which was followed by drying and harvesting of seeds. Primary transformants were selected in T_1_ generation using kanamycin (50 mg/L). The seeds from the putative transgenics were again selfed to obtain T_3_ progenies through T_2_.

Tobacco plant transformation was performed by leaf disc transformation method with minor modifications [45]. Small pieces of fully grown leaves were surface sterilized by 0.01% HgCl_2_ for 3–5 min, which was followed by 4–5 washes with sterilized water. The explants were then incubated in *Agrobacterium* (strain EHA105) cell (harboring pCAMBIA2300::AnnAt8 construct) suspension. Subsequently, the treated explants were dried with sterilized tissue paper and placed on full MS-Agar medium (with 2.0 mg/L BAP and 0.1 mg/L NAA) for cocultivation. After two days of cocultivation, the explants were transferred to the shoot initiation medium (MS-Agar medium with 2.0 mg/L BAP and 0.1 mg/L NAA) with kanamycin (125 mg/L) for transformed shoot selection and Cefotaxime (250 mg/L) to check *Agrobacterium* overgrowth. After four weeks on the shoot induction medium, newly formed shoots from the callus were transferred to shoot elongation (MS-Agar) medium. Further, the elongated shoots were transferred to a root initiation medium (MS-Agar medium with 0.1 mg/L NAA); both media contained Kanamycin for selection. Finally, the selected putative transgenic plantlets were kept for hardening in the growth room and then transferred to the greenhouse for further growth and analysis. The seeds from the putative primary transgenic plants were collected through self-pollination and were germinated on Kanamycin containing medium to screen the T_1_ plants, the confirmed plants were selfed to obtain T_2_ progeny.

### 2.4. Seed Germination Analysis in Salt and Osmotic Stress

For seed germination assay, *AnnAt8* overexpressing transgenic (T_3_ generation) and WT *Arabidopsis* seeds were surface sterilized by 1% sodium hypochlorite (NaOCl) for one min and then washed with sterile water 4–5 times. The seeds were transferred to the ½ MS-Agar medium and maintained in the growth chamber with a photoperiod of 8/16 h light/dark regime at 21 ± 2 °C room temp. For salt treatment, we used 100 mM of NaCl in half strength MS medium; for osmotic stress treatment, 250 mM sorbitol and for ABA treatment, 5 µM of ABA was used.

To test the seed germination rates in tobacco, T_2_ transgenic (T-14.3 and T-15.2) and WT (Control) seeds were surface sterilized in 75% ethanol for one min, which was followed by treatment with sodium hypochlorite (NaOCl) for ten min. After sterilization, seeds were washed 4–5 times with sterile water and transferred to the half strength MS + 0.8% Agar (HiMedia Lab. Pvt. Ltd. Mumbai, India) plates and maintained in a culture room with a photoperiod of 16 h light and 8 h dark at room temp 27 ± 1 °C. For salt treatment, we used 0, 100, 150 and 200 mM of NaCl in half strength MS-Agar medium. For inducing osmotic stress, 0, 300 and 400 mM sorbitol concentrations were used.

### 2.5. Seedling Stress Assay

For *Arabidopsis* seedling assays, *AnnAt8* overexpressing *Arabidopsis* transgenic lines and WT seeds were surface sterilized by NaOCl and were allowed to germinate in a growth chamber maintained at 21 ± 2 °C with a photoperiod of 8/16 h light/dark. After seven days of post-germination growth on half strength MS-Agar medium, WT and *AnnAt8* overexpressing seedlings were transferred to stress media and were grown on vertically placed agar plates. Seedlings of WT and transgenic lines were allowed to grow further on only half strength MS medium for control, and half strength MS media with 100 mM NaCl for salt stress and 250 mM sorbitol for osmotic stress respectively. For all experiments, except the NaCl treatment, seedlings were grown on semi-vertical agar plates.

To evaluate the stress tolerance of transgenic tobacco plants at the seedling stage, we exposed the transgenic and WT seedlings to different stress conditions for different time periods. For all the bioassays performed in the current study; we used T_2_ generation seeds. For seedling assay, T_2_ generation seeds of transgenic lines, and WT were surface sterilized by NaOCl and placed on half strength MS (without organics) + 0.8% Agar (HiMedia Lab. Pvt. Ltd. India). Seedlings were allowed to grow for ten days in a growth chamber maintained at 27 ± 1 °C with a photoperiod of 16/8 h light/dark. Ten days old seedlings of WT and transgenic lines were transferred on half strength MS-Agar medium with 100 and 200 mM NaCl for salt stress. Dehydration stress was given by 200 and 300 mM sorbitol in half strength MS-Agar medium. For photooxidative stress, 10 µM Methyl Viologen (MV) was used in half strength MS-Agar medium. For each experiment, seedlings were grown on horizontal agar plates.

### 2.6. Leaf Disc Senescence Assay

Fully grown leaves from two-month old tobacco plants were taken for leaf disc senescence assays. The leaf discs of 1 cm diameter were excised with the help of a cork borer and used further in stress treatments. Sorbitol (300, 400 and 500 mM conc) was used to induce dehydration stress. Similarly, NaCl solutions of 100, 200 and 300 mM concentrations were used for salt stress. Water was used as a control. The treatments were carried out in continuous light until identifiable differences were observed.

### 2.7. Biochemical Analyses: Determination of Chlorophyll Content and Lipid Peroxidation Assay

To measure the total chlorophyll content of seedlings and leaf discs, equal quantities of treated and non-treated samples were taken. The total chlorophyll content of seedlings and senescing leaf discs was estimated according to Hiscox and Israelstam [46]. Lipid peroxidation in the form of thiobarbituric acid reactive substances was estimated following the MDA estimation protocol [47].

### 2.8. Estimation of Proline Content in Transgenic and WT Tobacco Seedlings

Fifteen day-old seedlings of WT and transgenic lines were transferred onto half strength MS-Agar medium with 200 mM NaCl, and seedlings grown only on half strength MS-Agar medium were used as a control. After four days of NaCl treatment, seedlings were used for estimation of proline according to Bates *et al.* [48].

### 2.9. Real Time Expression Analysis of Stress-Inducible Genes in WT and Transgenic Tobacco Plants

For the evaluation of transcript levels of different stress marker genes, 15 day-old transgenic, and WT tobacco seedlings were incubated with half strength MS (control) and 200 mM NaCl (treatment) for 6 h and samples were collected for RNA isolation. Total RNA was extracted using TRIZOL (Invitrogen, Waltham, MA, USA). The first strand of cDNA was prepared from 2 µg of total RNA using MMLV Reverse Transcriptase (Clontech, Mountain View, CA, USA) in a reaction volume of 20 µL. For the real-time expression analysis, FAST START-SYBR MIX (Roche, Penzberg, Germany) was used. All the reactions were performed in Mastercycler^®^ 204 ep realplex4 (Eppendorf, Hamburg, Germany). PCR program used for all the amplifications was 95 °C for 10 min and 40 cycles of 95 °C for 15 s (denaturation), 57 °C for 30 s (annealing), 72 °C for 30 s (elongation). Primers used in the qPCR analysis are listed in Supplementary Table S3. Each sample was amplified in three independent biological replicates with three technical replicates. Relative gene expression was calculated according to the ΔΔC_T_ method [49]. *NtActin* was used as a reference gene to normalize gene expression in tobacco. ΔC_T_ for WT (untreated) was used as a control for the calculation of relative changes in expression of transgenic lines (untreated) and NaCl-treated WT and transgenic lines.

### 2.10. Subcellular Localization of AnnAt8 Protein

To check the subcellular localization of AnnAt8 protein, the ORF was reamplified with ORF2-F and ORF2-R primers harboring *Eco*RI and *Sma*I sites respectively. The amplified product was further digested and cloned in the pEGAD vector at the same sites, as a C-terminal fusion protein. Primers used in the amplification of ORF2 are listed in Supplementary Table S1. Further, the confirmed vectors were mobilized into *Agrobacterium tumefaciens* strain EHA105. The *Agrobacterium* cell (harboring the pEGAD-AnnAt8 construct) suspension was infiltrated in tobacco leaves (*Nicotiana tabacum* Linn cultivar Samsun) and GFP expression was visualized under the confocal microscope (Carl Zeiss LSM 710 NLO ConfoCor 3, Germany) at 480 to 515 nm wavelength after a 48 h incubation period, according to Kumar and Kirti [50].

### 2.11. Statistical Analysis

All the data was analyzed by the statistical analysis of variance (one-way ANOVA) by SIGMA PLOT, St. Louis, MO, USA ver. 11.0. 

## 3. Results

### 3.1. Molecular Analysis of Transgenic Arabidopsis and Tobacco Plants

**Figure 1:**
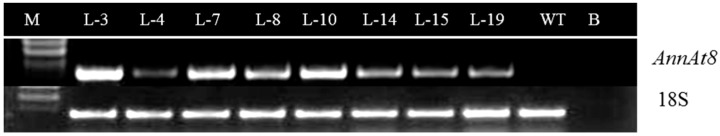
Expression analysis of *AnnAt8* by semi-quantitative RT-PCR in *AnnAt8* overexpressing transgenic *Arabidopsis* plants confirmed at T_1_ generation. WT is taken as a non-transgenic control, B is non-template control. 18S used as reference gene.

For the molecular and functional characterization of AnnAt8, *AnnAt8* overexpressing transgenic *Arabidopsis* and tobacco plants were generated through *Agrobacterium*-mediated genetic transformation of *Arabidopsis thaliana* ecotype Col 0 and *Nicotiana tabacum* L. cultivar Samsun. Transgenic *Arabidopsis* plants were generated by the standard floral dip method [44]. In T_1_ generation, putative transgenic plants were selected on half strength MS medium containing Kanamycin (50 mg/L), and the putative transformed plants were analyzed for the presence of transgene by CaMV35S and AnnAt8 gene-specific PCR (Supplementary Figure S1). Along with this, the T-DNA integration was cross confirmed by *NptII* gene specific PCR (data not shown)*.* Primer sequences used for the PCR analysis of transgenes are mentioned in the Supplementary Table S2. Further, on the basis of *AnnAt8* gene expression analysis in the T_1_ generation (L-3, L-4, L-7, L-8, L-10, L-14, L-15 and L-19) (Figure 1), two transgenic lines (L-3, L-7) were finally selected for detailed analysis in the T_3_ generation (obtained through selfing and cross checking).

**Figure 2:**
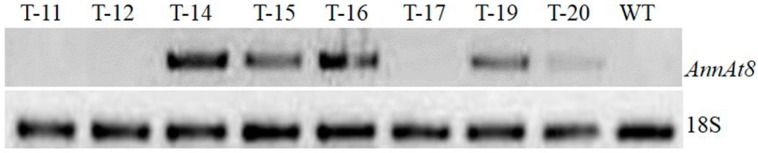
Expression analysis of *AnnAt8* in *AnnAt8* overexpressing transgenic tobacco plants by semi-quantitative RT-PCR. WT is taken as non-transgenic control. 18S was used as a reference.

Since the functional analysis of AnnAt8 in *Arabidopsis thaliana* transgenic showed the AnnAt8 involvement in abiotic stress tolerance, we proceeded to see whether *AnnAt8* can be used for the crop transformation purposes for the improving stress tolerance. Hence, tobacco was used as a model system, and we generated transgenic tobacco plants expressing *AnnAt8* in a constitutive manner and evaluated the plants for stress tolerance at different stages of growth. Tobacco transformation was done by standard leaf disc protocol [45]. In T_0_ generation, 16 independent putative transformants were analyzed for the presence of transgenes by gene-specific PCR using *NptII* (Supplementary Figure S2) and *AnnAt8* gene specific primers. Primer sequences used for the PCR analysis of transgene are mentioned in the Supplementary Table S2. Further, on the basis of *AnnAt8* gene expression analysis using semi-quantitative RT-PCR in putative transgenic plants of T_0_ generation (Figure 2), eight plants (T-11, 12, 14, 15, 16, 17, 19 and T-20) were selected for further analysis in T_1_ generation. Based on the expression analysis and stress assay performed in the T_1_ generation on T-11.1, T-12.1, T-14.3, T-15.2, T-17.1, T-19.1 and T-20.1, three transgenic lines were finally selected for detailed analysis (T-14.3, T-15.2, T-19.1) in the T_2_ generation. Transgenic lines T-14.3 and T-15.2 were used in all assays while line T-19.1 was also included in some experiments.

### 3.2. Enhanced Seed Germination of Arabidopsis Transgenic Plants under Stress Conditions

**Figure 3:**
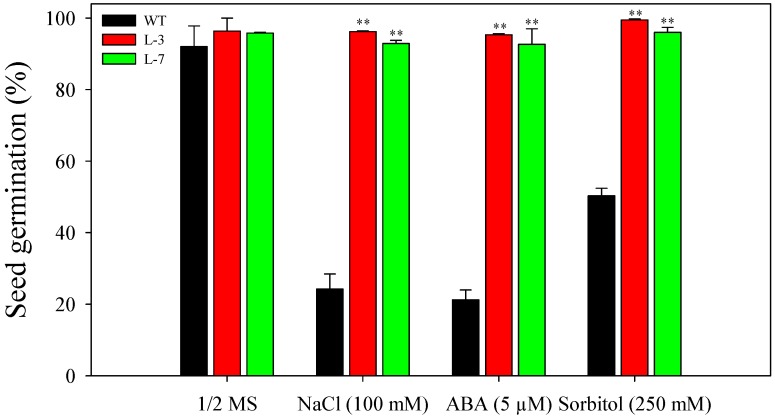
Seed germination of WT and *AnnAt8* overexpressing (OE) *Arabidopsis* plants under different stress conditions. Seed germination percentage of WT and *AnnAt8* OE *Arabidopsis* transgenic lines L-3 and L-7 under normal (half strength MS) and salt (100 mM), ABA (5 µM) and osmotic stress (sorbitol 250 mM). In each case, the observations were made on the third day after incubation under germination condition. For each biological replicate (n), 100 seeds of WT and transgenic lines were taken. Data represent the means of *n* = 3 ± SE. Two asterisks indicate that the mean values of transgenic lines were significantly higher than that of WT (control) when analyzed by one-way ANOVA (*p* < 0.01).

Seed germination rates of WT and *AnnAt8* overexpressing *Arabidopsis* seeds were checked under different stress treatments. On regular half strength MS medium, more than 90% of WT and transgenic seeds completed germination at day 3. In the case of NaCl (100 mM) treatment, WT showed only 20% seed germination, while transgenic lines showed more than 90% of seed germination. At 5 µM ABA, a strong inhibition was observed in the germination of WT seeds, and only 20% seed germination was observed. However, the transgenic lines showed more than 90% seed germination at this concentration of ABA. The osmotic stress condition generated by adding sorbitol in the medium showed comparatively less inhibition in seed germination; 50% of seed germination was observed in the case of WT at 250 mM sorbitol concentration while *AnnAt8* overexpressing (OE) lines showed more than 95% seed germination at the same concentration. In each case, observations were made on day 3 after initiation of the treatment (Figure 3).

### 3.3. Salt and Dehydration Stress Tolerance of AnnAt8 Transgenic Arabidopsis Plants

**Figure 4:**
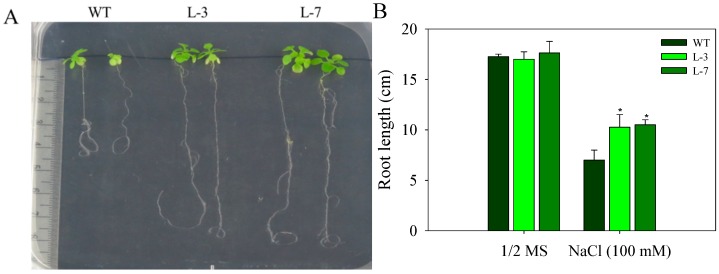
The growth of WT and *AnnAt8* overexpressing *Arabidopsis* plants under normal and salt (100 mM) stress conditions (**A**) Plant growth of WT and *AnnAt8* expressing *Arabidopsis* transgenic lines L-3 and L-7 under NaCl (100 mM) stress. Three replicates were used per treatment, and the experiment was repeated thrice. (**B**) Root length of WT and AnnAt8 expressing *Arabidopsis* transgenic plants under normal and salt stress. In each case, observations were made after 44 days from germination. For root length measurement, average value for 15 plantlets of WT and transgenic lines was taken for each biological replicate (n). Data represent means of *n* = 3 ± SE. One asterisk indicates that the mean values of transgenic lines were significantly higher than that of WT (control), when analyzed by one-way ANOVA (*p* < 0.05).

Different growth parameters like root growth and fresh weight were assayed to observe the effect of stress conditions on the growth of seedlings and young plants of WT and *AnnAt8* OE lines L-3 and L-7. After 44 days of growth on NaCl (100 mM) medium, we observed that NaCl caused less inhibition on the growth of *AnnAt8* expressing plants in comparison to WT plants (Figure 4A,B).

**Figure 5:**
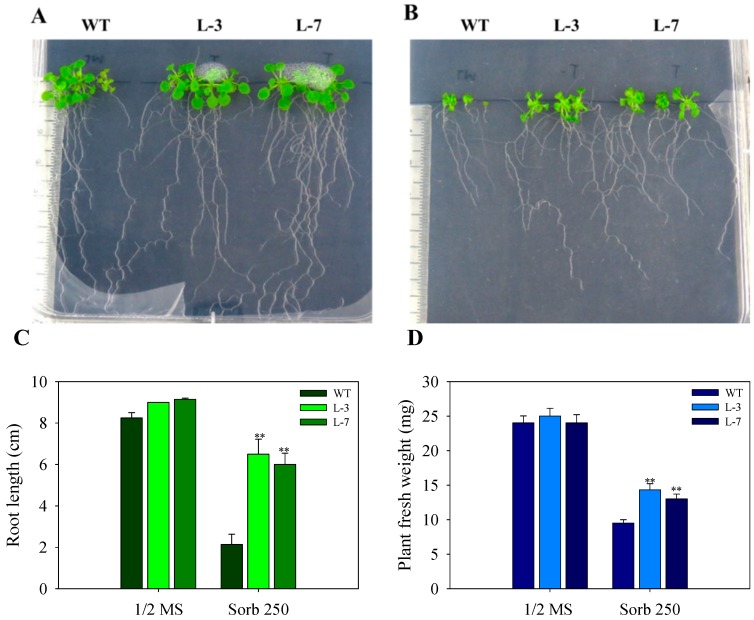
Effect of osmotic stress on the growth of WT and *AnnAt8* expressing *Arabidopsis* plants. (**A**,**B**) Root and shoot growth of plants under normal (half strength MS) and osmotic stress (sorbitol 250 mM), respectively. In each case, the observations were made on day 28 after germination. Three replicates were used per treatment, and the experiment was repeated thrice; (**C**) Root length of WT and transgenic lines L-3 and L-7 under normal and osmotic stress conditions; (**D**) Fresh weight of WT and transgenic lines under normal and osmotic stress conditions. For root length and fresh weight data, observations were made on day 28 and for each replicate (n) the average values for 15 plants of WT and transgenic lines were taken. Data represent means of *n* = 3 ± SE. Two asterisks indicate that the mean values of transgenic lines were significantly higher than that of WT (control) when analyzed by one-way ANOVA (*p* < 0.01).

As it is known that NaCl imposes osmotic stress as well as ion toxicity on the plants, we used sorbitol in half strength MS-Agar medium to check for the osmotic stress effect and monitored the plant growth at different time points. At the early stage of observation after 15 days of growth, we found that the growth of WT seedlings was more hampered than *AnnAt8* expressing lines L-3 and L-7 (data not shown). We also noted that the growth inhibition caused by 250 mM Sorbitol was less than that of 100 mM NaCl treatment (data not shown). Another observation made at day 28 showed that the transgenic plants not only survived under high osmotic stress but also showed better root growth, with about threefold increase as compared to WT (Figure 5), while in the normal growth condition, a negligible difference was seen between the growth of WT and transgenic plant. With the advancement of time, we observed that the transgenic plants showed better adaptation to the dehydration stress while strong inhibition was observed in the growth of WT plants.

**Figure 6:**
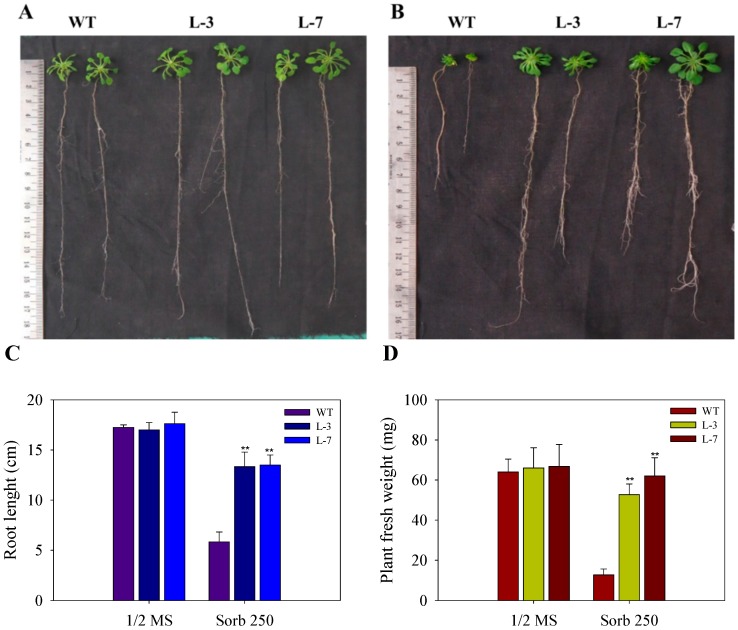
Effect of osmotic stress on the growth of WT and transgenic *Arabidopsis* plants (**A**,**B**) growth of plant under control (half strength MS) and osmotic stress (sorbitol 250 mM) conditions, respectively. Three replicates were used per treatment and the experiment was repeated thrice. (**C**) Root length of WT and transgenic L-3 and L-7 under normal and osmotic stress conditions; (**D**) Fresh weight of plants. In each case observation was made on day 44 after germination. For root length data, the average values of 15 plants of WT and transgenic lines were taken for each biological replicate (n). Data represent means of *n* = 3 ± SE. Two asterisks indicate that the mean values of transgenic lines were significantly higher than that of WT (control) when analyzed by one-way ANOVA (*p* < 0.01).

An observation made at day 44 showed that the growth of WT was completely inhibited while the transgenic plants maintained their normal growth under osmotic stress (250 mM Sorbitol). Root length and fresh weight measurements of WT and lines L-3, L-7 were also performed (Figure 6C,D). We measured the lateral root density of plants and found that the lateral root density was higher in the transgenic plants compared to the WT under osmotic stress (data not shown). Fresh weight of plants was also measured at different time points of stress treatment (Figure 5D and Figure 6D).

### 3.4. Osmotic and Salt Stress Tolerance of AnnAt8 Transgenic Tobacco Plants

**Figure 7:**
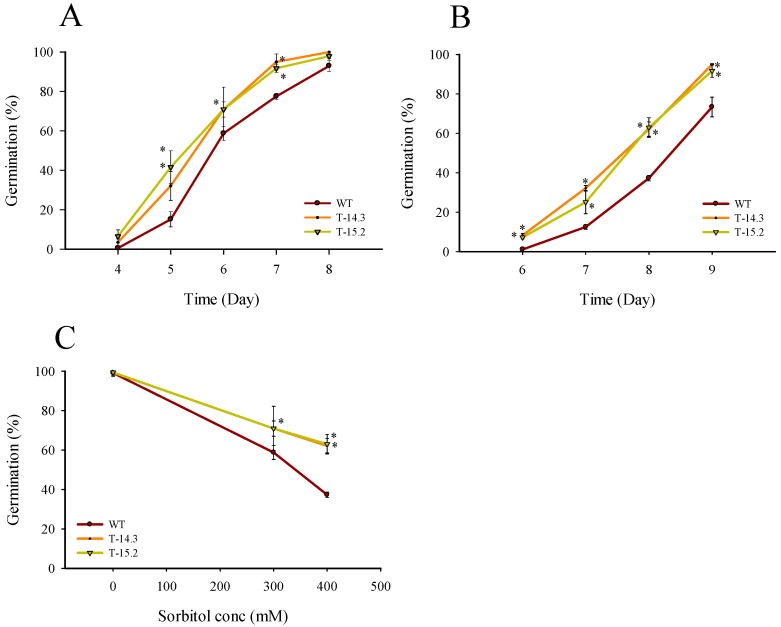
Seed germination rates of WT and *AnnA8* overexpressing transgenic tobacco plants under osmotic stress condition. (**A**, **B**) are day wise percentages of seed germination respectively on 300 and 400 mM sorbitol; (**C**) Sorbitol dose response on the seed germination, percentage value of seed germination on 0, 300 and 400 mM sorbitol were taken on day 4, 7 and 8, respectively, from the date of plating on the media, for each biological replicate (n) 150 seeds of WT and transgenic lines were taken. Data represent means of *n* = 3 ± SE. An asterisk indicates that the mean values of transgenic lines were significantly higher than that of WT (control) when analyzed by one-way ANOVA (*p* < 0.05).

We evaluated the tolerance of *AnnAt8* transgenic tobacco plants under osmotic stress at different stages of plant growth. Sorbitol was used in this study to induce osmotic stress. Seed germination, seedling, and mature plant stages were observed in the present study. The germination percentages of WT and transgenic lines were almost similar under control condition (without sorbitol), (Figure 7C). More than 95 percent of the seeds of the WT and transgenic lines germinated after 3 days of plating on the control medium, while in 300 and 400 mM sorbitol containing half strength MS media, the overall germination was delayed in WT and transgenic lines, in comparison to the control condition. At 300 mM sorbitol concentration, the seed germination started on day 4, and it continued up to the day 8 of observation. The day wise observations showed that the transgenic lines showed a significantly higher percentage of germination compared to WT (Figure 7A). Similarly, on 400 mM sorbitol medium, the day wise percent germination of transgenic seeds was higher than the WT seeds (Figure 7B).

**Figure 8:**
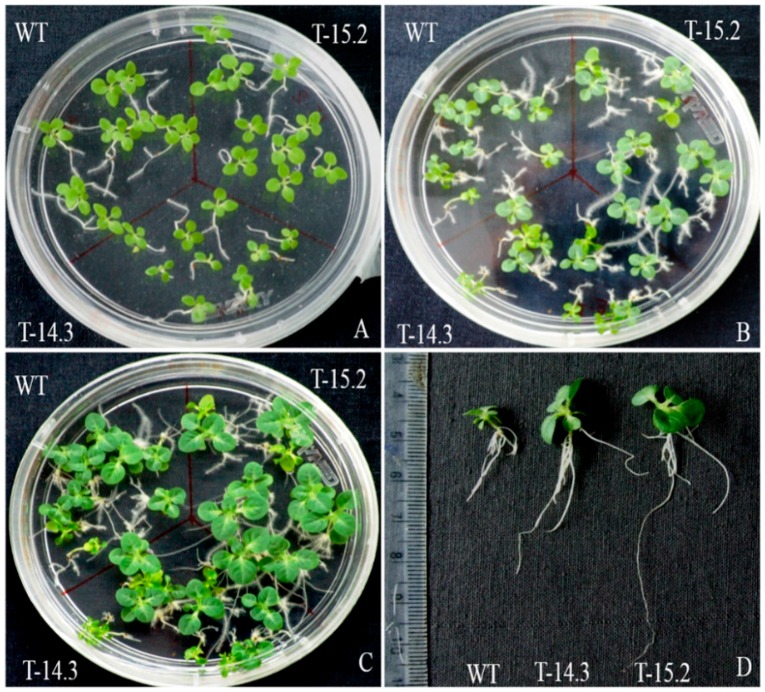
Seedling growth of WT and *AnnAt8* transgenic tobacco plants under osmotic stress. (**A**) Seedlings on day 15 after transfer on half strength MS medium; (**B**,**C**) seedlings on day 35 and 63, respectively after transfer on half strength MS medium supplemented with 300 mM of sorbitol; (**D**) comparison of root and shoot growth after two months of osmotic stress (300 mM sorbitol). Three replicates were used per treatment and the experiment was repeated thrice.

**Figure 9:**
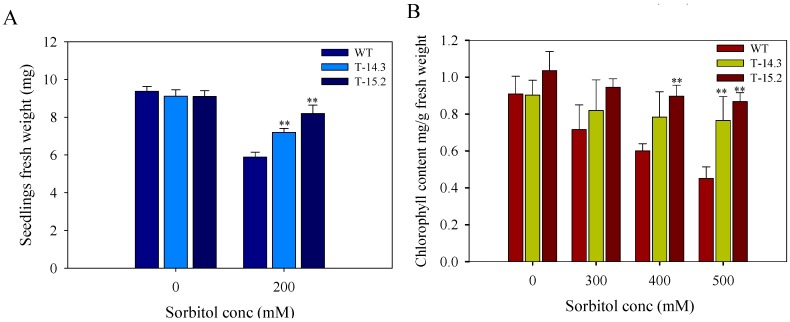
Fresh weight and estimation of total chlorophyll content of WT and transgenic tobacco plants under osmotic stress condition (**A**) Seedling weight under normal and osmotic stress after 26 days of growth. For seedling weight, 10 seedlings for each biological replicate (n) were used, (**B**) Total chlorophyll content of WT and transgenic lines on 0, 300, 400 and 500 mM sorbitol solution. Data represent means of *n* = 3 ± SE. Two asterisks indicate that the mean values of transgenic lines were significantly higher than that of WT when analyzed by one-way ANOVA (*p* < 0.01).

For seedling stress assay, 10 day-old seedlings were transferred to the half strength MS medium with different concentrations (100–200–300 mM) of sorbitol. No differences were observed between the WT and the transgenic lines at 100 mM sorbitol concentration. At 200 mM, there were significant differences in the root and shoot growth of transgenic seedlings compared to WT (data not shown). Increased root and shoot growth was observed in the transgenic lines with enhanced root branching. At 300 mM sorbitol concentration, the seedling growth of WT and transgenic plants was slow in the beginning. However, the transgenic lines resumed active growth with the passage of time showing overall enhanced growth compared to the WT (Figure 8). A significant difference in the seedlings biomass was also observed under osmotic stress with a higher fresh weight of transgenic lines in comparison to WT (Figure 9A).

Leaf disc senescence assay was performed to evaluate the total chlorophyll content of WT and transgenic lines under normal and stress conditions; leaf discs from the mature leaves of two-month-old transgenic and WT plants were taken for analysis. At 300 mM concentration, we did not find any significant difference in chlorophyll content of WT and transgenic lines. At 400 mM concentration, one transgenic line showed significantly higher chlorophyll content compared to WT. The total chlorophyll content of both transgenic lines was significantly higher than WT at 500 mM sorbitol concentration (Figure 9B).

**Figure 10:**
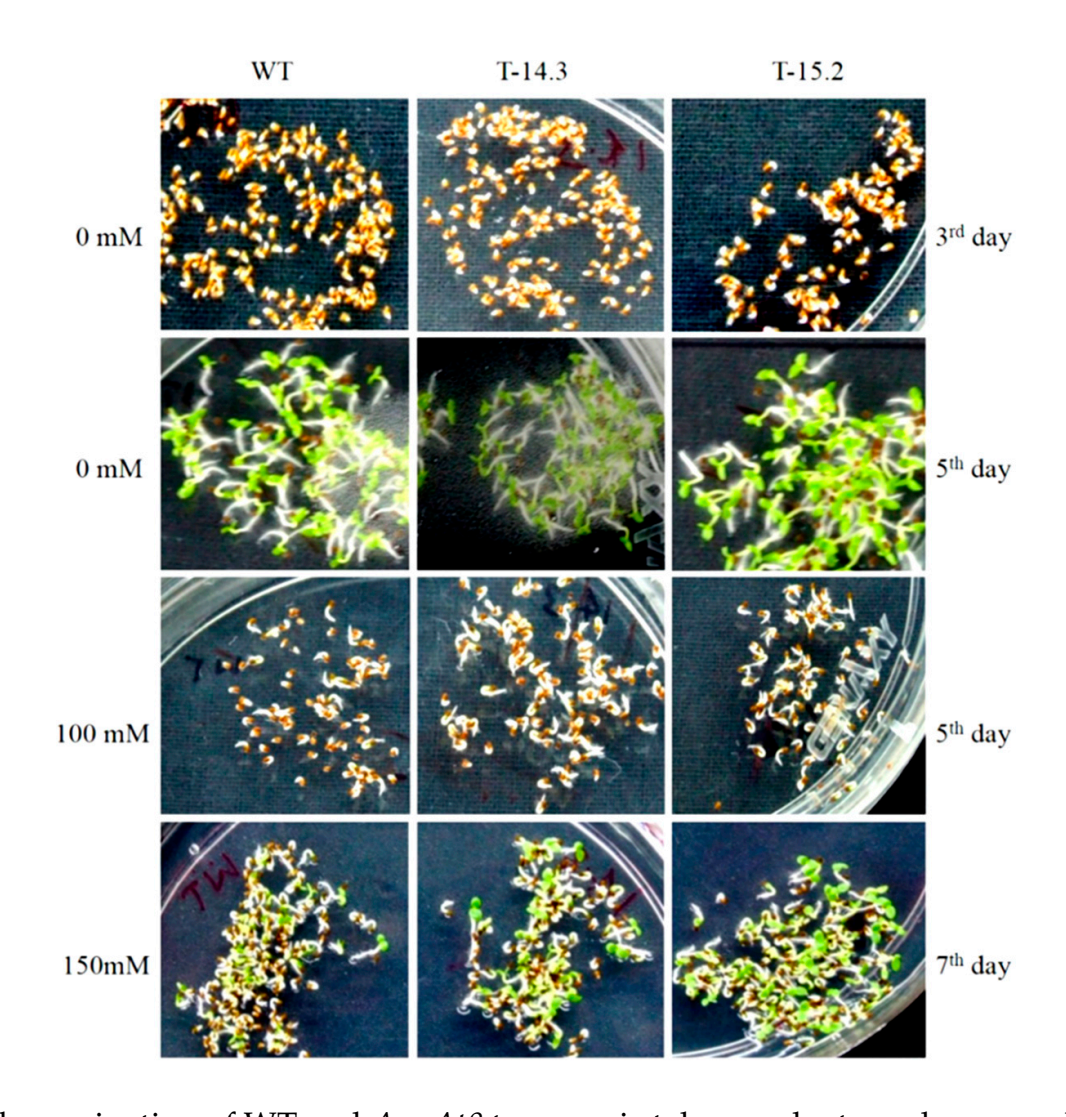
Seed germination of WT and *AnnAt8* transgenic tobacco plants under normal and salt stress conditions. There were no differences in seed germination rates of WT and transgenic lines on half strength MS medium. On NaCl (100 and 150 mM concentrations) media, transgenic lines showed a higher rate of seed germination compared to WT. Three replicates were used per treatment and the experiment repeated three times.

**Figure 11:**
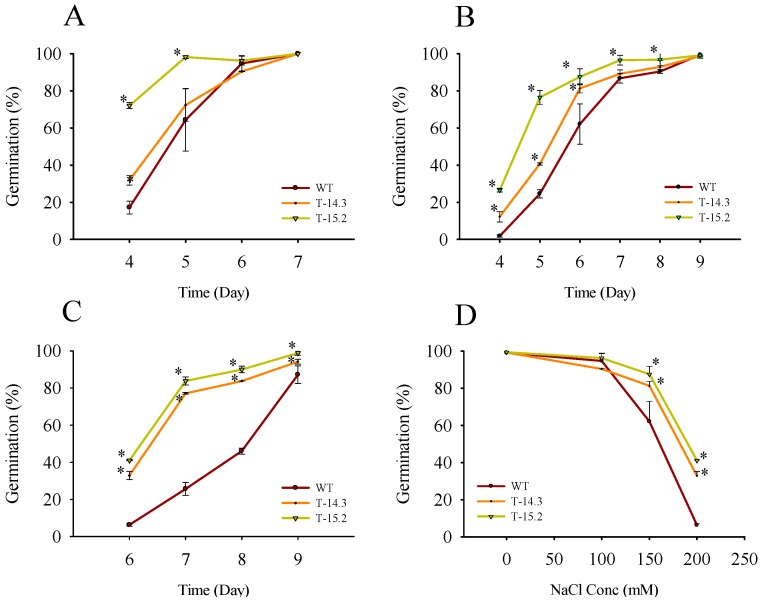
Seed germination rates of WT and *AnnAt8* overexpressing transgenic tobacco plants under salt stress. (**A**–**C**) are day wise seed germination percentages on 100, 150 and 200 mM NaCl concentrations, respectively; (**D**) NaCl dose response on seed germination. For each biological replicate (n) 150 seeds of WT and transgenic lines were taken. Data represent means of *n* = 3 ± SE. An asterisk indicates that the mean values of transgenic lines were significantly higher than that of WT, when analyzed by one-way ANOVA (*p* < 0.05).

As salt tolerance in crop plants is of commercial significance, we studied the response of *AnnAt8* expressing transgenic tobacco plants towards sodium chloride treatment. In the case of seed germination, three different concentrations of sodium chloride (100, 150 and 200 mM) were used to check the germination rates. We observed a delay in the germination of WT and transgenic seeds on sodium chloride containing germination media. On the germination medium without NaCl, seeds of all the transgenic lines including WT, almost completed their germination on day 3 (Figure 10). Transgenic lines showed higher germination rates compared to WT at NaCl concentration of 100, 150 and 200 mM (Figure 11). At 200 mM conc, about 75%–85% seeds of the transgenic lines germinated by day 7, while the seed germination of WT was only around 25%. Percent germination observed on each day showed a significant difference between WT and transgenic lines (Figure 11A, B). At 200 mM NaCl, we found a very significant difference in the germination percentage from day 6 onwards itself (Figure 11C). The germination percentages of WT and transgenic lines on control media at day 4, and on the stress media (with 100, 150 and 200 mM NaCl concentration respectively) at day 6 were used to check the NaCl doses response (Figure 11D).

**Figure 12:**
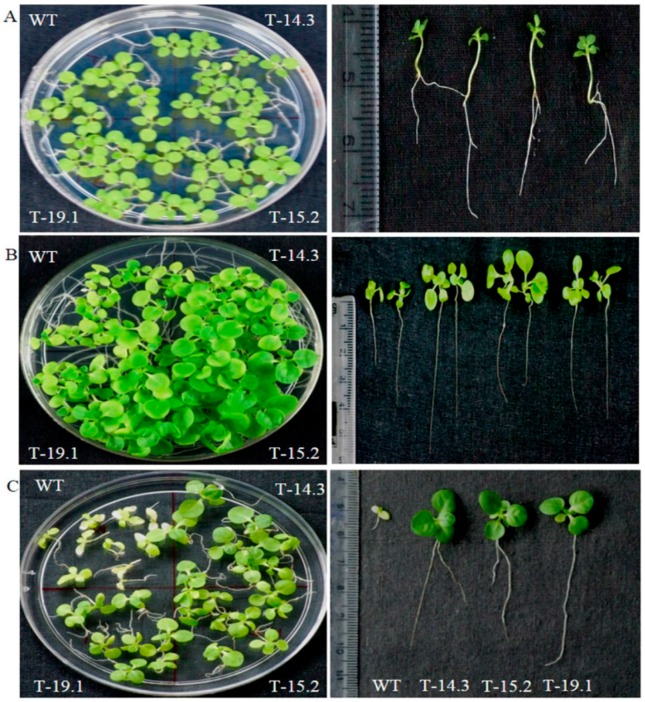
Seedling growth under salt stress. (**A**) Seedlings after 18 days of transfer on half strength MS medium; (**B**) seedlings of WT and *AnnAt8* transgenic lines after 56 days of growth on 100 mM NaCl; (**C**) seedlings of WT and transgenic lines after 42 days of growth on 200 mM NaCl. Three replicates were used per treatment and the experiment repeated three times.

**Figure 13:**
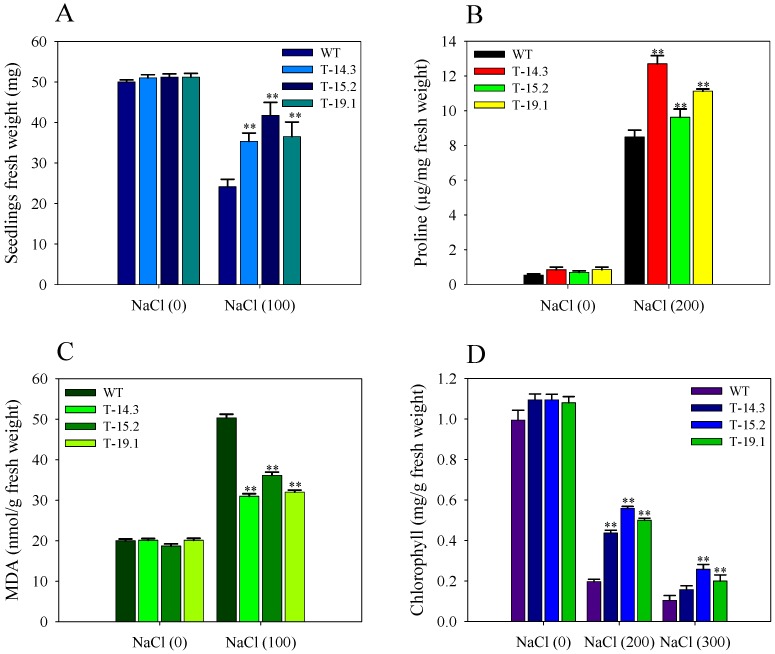
Seedling biomass of *AnnAt8* transgenic tobacco plants under salt stress and measurement of chlorophyll, malondialdehyde (MDA) and proline content under salt stress. (**A**) Fresh weight of transgenic and WT seedlings after nine weeks of growth on half strength MS (with 0 and 100 mM NaCl). For seedling weight average, 10 seedlings for each biological replicate (n) were used; (**B**) Proline content of transgenic and WT seedlings with (200 mM NaCl) and without salt treatment; (**C**) MDA content of transgenic and WT seedlings with (100 mM NaCl) and without salt treatment (**D**) Total chlorophyll content of leaf discs from WT and transgenic tobacco plants with and without NaCl treatment. The data represent the means of *n* = 3 ± SE. Two asterisks indicate that the mean values of transgenic lines were significantly higher than that of WT when analyzed by one-way ANOVA (*p* < 0.01).

Initially the seedling growth of WT and transgenic plants was normal on 100 mM concentration. With the advancement of time, WT showed retardation in the growth. After eight weeks of growth on 100 mM NaCl medium, transgenic lines showed better root and shoot growth compared to WT (Figure 12B). At 200 mM concentration, both WT and transgenic lines showed slow growth in the initial stages. Subsequently, the transgenic seedlings showed better adaptation in root and shoot growth compared to WT (Figure 12C). Seedling weight measurement of NaCl-treated seedlings also showed a higher fresh weight of transgenic lines in comparison to WT (Figure 13A). Additionally, to check the tolerance at a higher level of NaCl concentration, seedlings were transferred on 300 mM NaCl containing half strength MS-Agar medium for 16 days. A uniform growth inhibition and bleaching were observed in WT and transgenic lines. After stress treatment, WT and transgenic seedlings were transferred to the recovery medium and we observed that transgenic seedlings showed 90% recovery with a quick resumption of growth, while only 50% of WT seedlings did so (data not shown).

**Figure 14:**
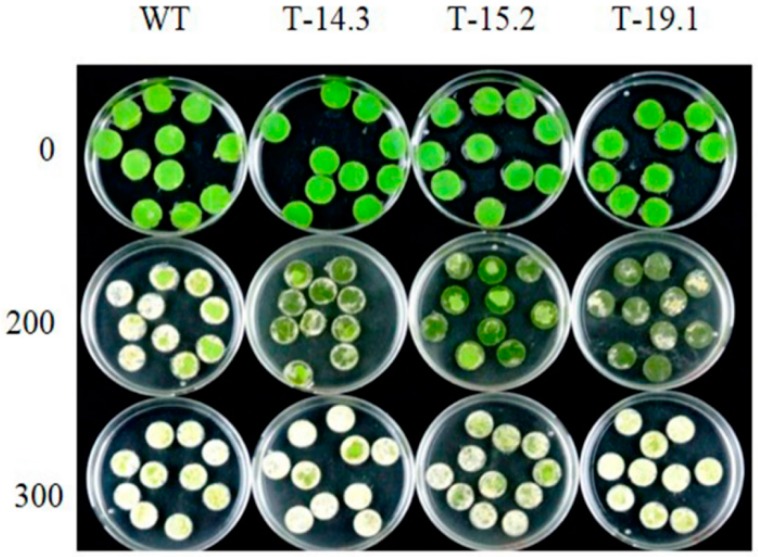
Leaf disc senescence assay under salt stress. Leaf discs from fully expanded leaves of two-month-old WT and *AnnAt8* transgenic tobacco plants were floated on NaCl solution for the stress treatment. The treatments were carried out in continuous light until identifiable differences were observed. Leaf discs of the WT plants bleached out faster in the stress medium (0, 200, 300 mM NaCl). However, the chlorophyll loss was slow in the transgenic lines. Three replicates were used per treatment, and the experiment was repeated at least three times and similar findings were observed.

Proline estimation showed a several-fold increase in proline content of the transgenic and WT seedlings after four days of NaCl treatment. However, the proline content of the transgenic seedlings was significantly higher than WT (Figure 13B). Lipid peroxidation assay, performed with the NaCl-treated transgenic and WT seedlings also showed lower lipid peroxidation in the transgenic plants compared to the WT plant (Figure 13C). Total chlorophyll contents of WT and transgenic lines were estimated after a leaf disc senescence assay and WT showed less total chlorophyll content in comparison to transgenic lines (Figure 13D and Figure 14).

### 3.5. Alleviation of Methyl Viologen Stress in AnnAt8 Transgenic Tobacco Plants

**Figure 15:**
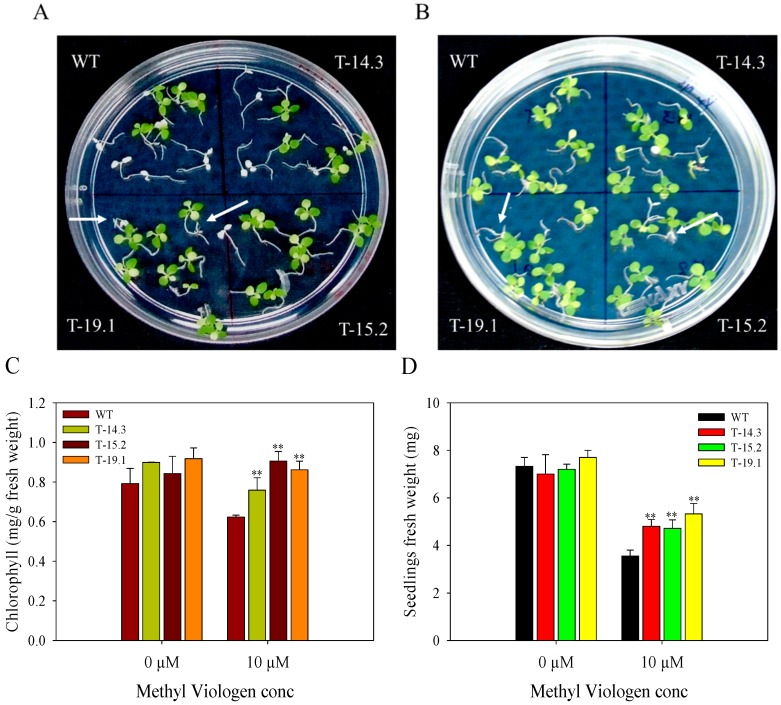
Seedling growth of *AnnAt8* transgenic tobacco plants under Methyl Viologen (MV) stress. (**A**) Seedlings on the 18th day after transfer on stress medium, transgenic lines showed better adaptation with the formation of new adventitious roots while WT showed bleaching and complete inhibition of growth; (**B**) Seedlings on recovery medium after 14 days of MV stress; transgenic lines showed fast recovery compared to WT. (White arrows indicate adventitious root formation in the seedlings). All experiments were performed in triplicate and repeated three times; (**C**) Total chlorophyll content of the recovered seedlings after a prolonged exposure on MV (10 µM) containing half strength MS medium; (**D**) fresh weight of *AnnAt8* transgenic and WT seedlings with and without MV (10 µM) treatment. Seedlings were treated with MV for 12 days and then transferred to the recovery medium for nine days, seedlings grown on half strength MS medium without MV were used as a control; for seedling weight, 10 seedlings from each biological replicate (n) were used and data represent means of *n* = 3 ± SE. Two asterisks indicate that the mean values of transgenic lines were significantly higher than that of WT when analyzed by one-way ANOVA (*p* < 0.01).

To check the photooxidative stress tolerance in transgenic plants, we subjected the WT and transgenic seedlings to 10 µM Methyl Viologen (MV) on half strength MS medium. We observed that the green tissue that directly came in contact with the medium bleached completely in 3–4 days of exposure (data not shown). The seedlings in which the green tissues did not come into contact with the medium also bleached but at a slower rate, and continued their growth with new adventitious root formation. The WT did not show any new root growth and adventitious root formation up to 18 days of observation (Figure 15 A). For recovery assay, seedlings were transferred to half strength MS-Agar medium after 14 days of MV stress. Transgenic seedlings showed faster recovery compared to the WT seedlings (Figure 15B). After recovery, the total chlorophyll contents of the transgenic lines were higher in comparison to WT (Figure 15C). Seedling weight after 12 days of treatment with MV and then nine days of recovery on half strength MS also showed higher fresh weight of transgenic lines in comparison to WT (Figure 15D).

### 3.6. Enhanced Tolerance of AnnAt8 Transgenic Tobacco Plants to Osmotic Stress Condition

**Figure 16:**
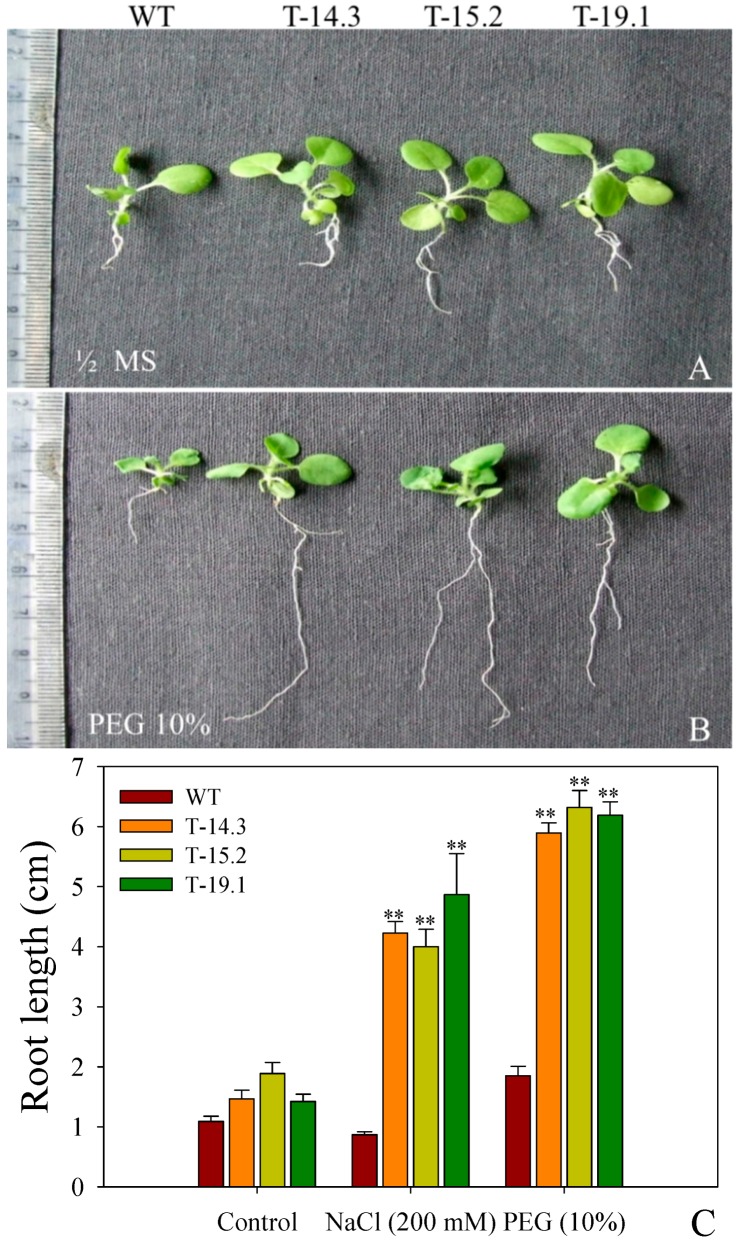
Seedling growth under salt and dehydration stress. (**A**, **B**) Seedlings of WT and *AnnAt8* overexpressing transgenic tobacco plants after five weeks of growth on half strength MS and 10% polyethylene glycol PEG; (**C**) Root lengths of WT and AnnAt8 transgenic seedlings after five weeks of growth on half strength MS, 200 mM NaCl and 10% PEG, respectively, for each biological replicate (n) 15 seedlings of WT and transgenic lines were taken. Data represent means of *n* = 3 ± SE. Two asterisks indicate that the mean values of transgenic lines were significantly higher than that of WT (control) when analyzed by one-way ANOVA (*p* < 0.01).

To check the effect of simulated drought condition on the seedling growth of WT and transgenic plants, 10% Polyethylene glycol (PEG) 4000 in half strength MS medium was used and half strength MS medium without PEG was used as a control. Initially, both WT and transgenic seedlings showed slow root and shoot growth on the PEG containing medium. The transgenic seedlings showed better adaptation at later stages of growth, with better root and shoot growth compared to WT (Figure 16).

### 3.7. Enhanced Root Growth of AnnAt8 Transgenic Tobacco Seedlings under Salt and Dehydration Stress

Evidence of better root growth of seedlings under salt and dehydration stresses was provided by carrying out statistical analysis of root growth of the seedlings after five weeks of stress treatment (Figure 16C).

### 3.8. Expression Analysis of Stress Related Genes in Transgenic and WT Tobacco Seedlings

**Figure 17:**
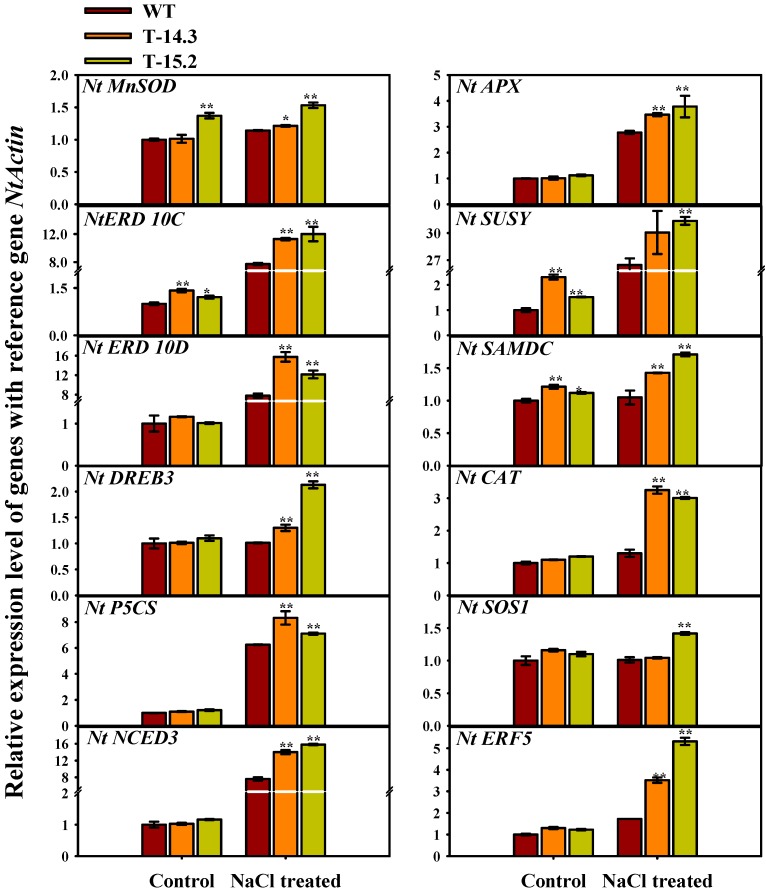
Real-time expression analysis of stress responsive genes in WT and *AnnAt8* transgenic tobacco seedlings before and after salt treatment to the seedlings, data represent means of *n* = 3 ± SE. An asterisk indicates that the mean values of transgenic lines were significantly higher than those of WT when analyzed by one-way ANOVA at *p* < 0.05 and two asterisks at *p* < 0.01.

Since the AnnAt8 transgenic lines showed enhanced tolerance to several stresses as detailed earlier, we studied the co-expression of some stress tolerance related genes, whose involvement in stress tolerance was demonstrated in previous reports. Real-time RT-PCR analysis was carried out for stress-related genes, which showed co-expression with AnnAt8 (Figure 17). Under stress conditions, the expression of *AnnAt8* in transgenic tobacco was accompanied by a significant upregulation of *NtMnSOD* (Manganese superoxide dismutase), *NtERD10C* (early response to dehydration 10C), *NtERD10D* (early response to dehydration 10D), *NtDREB3* (dehydration-responsive element binding protein), *NtP5CS* (Δ^1^pyrolline-5-corboxylate synthetase), *NtNCED3* (9-cis-epoxy carotenoid dioxygenase), *NtAPX* (Ascorbate peroxidase), *NtSUSY* (Sucrose synthase), *NtSAMDC* (S-adenosylmethionine decarboxylase), *NtSOS1* (salt overly sensitive 1), *NtCAT* (Catalase), and *NtERF5* (Ethylene responsive factor 5) transcripts (Figure 17). Both the high expression transgenic lines (T-14.3 and T-15.2) with enhanced stress tolerance showed higher expression of the stress related genes under NaCl treatment; the expression of these genes was always higher in these lines in comparison to WT under NaCl treatment (Figure 17).

### 3.9. Subcellular Localization of GFP-Tagged AnnAt8 Protein in Tobacco Cells

**Figure 18:**
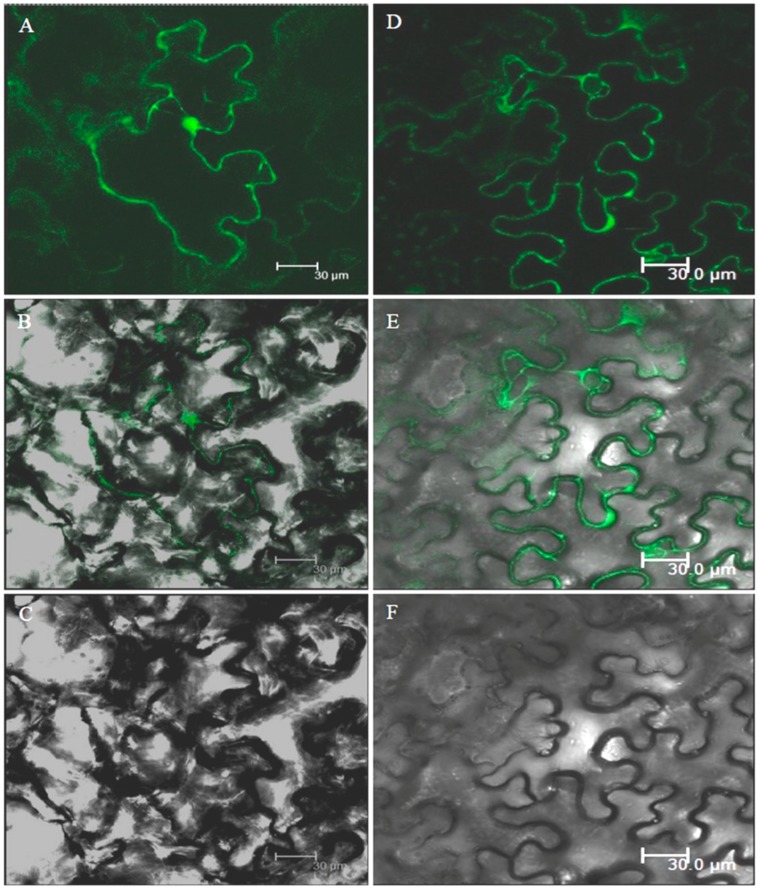
Subcellular localization of GFP-AnnAt8 by transient expression in tobacco leaf epidermal cells. (**A**–**C**) Subcellular localization of GFP control; (**D**–**F**) Subcellular localization of GFP-tagged AnnAt8.

To examine the subcellular localization of GFP-AnnAt8, 35Spro::GFP-AnnAt8 and an empty vector control (35Spro::GFP) were introduced individually into *Agrobacterium tumefaciens* EHA105. For the transient gene expression, tobacco leaf was infiltrated with the *Agrobacterium* cells. Then the tobacco leaf epidermal cells were observed for transient expression of the GFP Tagged protein under the confocal microscope. The fluorescence signal of the GFP control was observed all over the cell whereas the localization of the GFP-AnnAt8 fusion protein driven by the 35S promoter was observed at the cell periphery, but not in the nucleus of tobacco leaf epidermal cells (Figure 18).

## 4. Discussion

*Arabidopsis* has eight annexin genes in its genome and the expression analysis of all these genes under stress treatments has been reported earlier [31]. Few members (AnnAt1, AnnAt2, and AnnAt4) of *Arabidopsis* annexin family are well known for their role in abiotic stress tolerance [14,20,21]. Recently, AnnAt5 has been reported for its role in pollen development [16]. In an expression analysis, a very high level increase in *AnnAt8* transcripts was observed under salt and dehydration stresses in *Arabidopsis* seedlings [31]. However, the level of expression of a gene may not always lead to a positive or direct link to a particular phenotype. In the case of *AnnAt4*, the overexpression lines became more sensitive than WT under drought and salt stress treatments, and the *AnnAt4* mutant exhibited less sensitivity than WT to similar stress conditions [14]. Consequently, characterization of the annexin genes through expression in transgenic plants is necessary for linking their possible role in stress responses. In the present study, we generated transgenic *Arabidopsis* and tobacco plants expressing *AnnAt8* in a constitutive manner and assayed them for stress tolerance at different stages of the plant growth and development.

### 4.1. AnnAt8 Is Involved in Salt and Dehydration Stress Tolerance

*Arabidopsis* transgenic plants showed tolerance to NaCl stress at seed germination stage and further at seedling growth stage. Corroborating the *Arabidopsis* results, our observations in tobacco indicated that the seeds of *AnnAt8* transgenic tobacco plants germinated well under mild to higher concentrations of sodium chloride in the germination medium. Similarly, the transgenic tobacco seedlings also showed better survival and growth up to 300 mM NaCl. This indicates that AnnAt8 not only helps in seed germination but also in the further growth of the seedlings in salt stress conditions. The importance of seed germination under salinity cannot be underestimated as seeds fail to germinate in this condition. This is a major problem in saline soils and the plant stand after seed sowing would be poor. Seed germination was reduced under osmotic stress in WT and *AnnAt8* transgenic plants. However, the germination percentage of the transgenic seeds was significantly higher in comparison to WT plants in *Arabidopsis* and tobacco. But these differences in seed germination rates were less, compared to those observed in cases of salt stress given to *Arabidopsis* and tobacco seeds, which could be due to the additional ion (Na^+^) toxicity imposed during salt stress. The expression of AnnAt8 appears to ameliorate salt stress in the seed germination stage. Previous reports [14,21] showed similar beneficiary effects of annexin in salt stress at the seed germination stage. As salt stress has osmotic as well ion toxicity effects on the cell, there is a strong possibility of the involvement of AnnAt8 in defense against dual effects caused by salt stress at the germination and seedling stages.

Recent reports suggest that maize annexins and AnnAt1 can mediate the Ca^2+^ flux across the plasma membrane [26,27,28,29,51]. This Ca^2+^ modulation property of annexin might play an important role in the regulation of various stress-responsive pathways. Membrane binding and Ca^2+^ binding are important properties of annexin proteins, which are probably responsible for membrane integrity and modulation of the various abiotic stress responses, and the up-regulation of stress associated genes in *AnnAt8* transgenic plants. Ionic stress causes membrane damage, which can be due to high rates of membrane lipid peroxidation and we observed a lower level of lipid peroxidation in tobacco transgenic lines compared to WT, which shows that AnnAt8 has a role in membrane protection.

Root elongation is an adaptive mechanism during salt and drought stress. During salt and drought stress, roots are directly affected and a plant can sustain its shoot growth by maintaining normal root growth, by increasing water and nutrient uptake. We also found similar results in salt, dehydration and drought stress treatments. The transgenic *Arabidopsis* and tobacco seedlings grown on different stress media and the observations made at different time points indicate that AnnAt8 has an additive role in the normal adaptive mechanism of the seedlings under salt and dehydration stresses. This helps seedlings to maintain the integrity of cells and tissues during the early phase of stress, during the later stages of stress response, leading to better root growth in *AnnAt8* transgenic seedlings compared to WT.

### 4.2. AnnAt8 Is Involved in Alleviation of Methyl Viologen Induced Stress

Methyl Viologen (MV) stress inhibits the photosynthesis by interrupting regular electron flow in PSII causing formation of free radicals (peroxide) leading to membrane damage and ion leakage [52]. *AnnAt8* transgenic tobacco plants showed tolerance to MV at seedling stage; they not only survived on MV containing medium for a longer duration but also showed new adventitious root formation, which was not observed in WT seedlings (Figure 15A,B). *AnnAt8* transgenic showed reduced lipid peroxidation compared to WT (data not shown). Lipid peroxidation causes membrane damage, which leads to ion leakage. The higher chlorophyll content of MV stressed *AnnAt8* transgenic seedlings is also in support of stress alleviating effect of AnnAt8. Seedling biomass of transgenic tobacco lines was also higher than WT in MV stress. It suggests that AnnAt8 helped transgenic seedlings not only in surviving the MV stress, but also in maintaining their growth under MV-induced stress. A recent report also suggests that AnnBj3 of mustard plays a role in the alleviation of MV-mediated photooxidative stress in *Arabidopsis* [53]. The mechanism behind the AnnAt8 mediated MV stress tolerance needs to be studied further.

### 4.3. Localization Patterns of AnnAt8

Subcellular localization of GFP-AnnAt8 showed that the distribution of GFP-AnnAt8 fusion protein is peripheral in the cell and close to the membrane. Our observations are in line with the previous reports, which support annexin localization to the cell peripheral region [21,22,54]. We have not observed any nuclear localization in the present case. However, there are reports which support the distribution of some annexins to the nucleus and other organelles as well [55,56].

### 4.4. How Does AnnAt8 Respond to Abiotic Stress?

Co-expression studies of different stress responsive genes performed in *Arabidopsis* annexin knockout mutants and overexpressing transgenic mutants showed their altered expression under normal and stress conditions [14,29,51]. Expression studies of stress responsive genes performed on the WT and *AnnAt8* transgenic tobacco seedlings also showed an altered expression of different stress-responsive genes under normal and stress conditions.

Drought and salt stress responses overlap each other in many ways. Drought is mainly composed of osmotic stress whereas NaCl exerts osmotic as well as Na^+^ toxicity, which is controlled by a separate signaling pathway known as SOS (salt overly sensitive) [57]. Plants respond to these stress conditions with a change in its transcriptome. Genes expressed during salt and drought stress show an overlap and expression of these genes, regulated by many transcription factors which are common to both stresses.

Comparatively higher levels of transcripts of different stress responsive genes in *AnnAt8* transgenic lines compared to WT under salt stress condition show that AnnAt8 is associated with the differential regulation of these genes directly or indirectly. DREB proteins have been very well characterized for their role in drought tolerance [58]. *NtDREB3*, a member of the DREB family, showed a higher transcript level in *AnnAt8* transgenic lines compared to the WT seedlings in NaCl stress. This increase in transcript levels could be one reason for salt and dehydration tolerance responses exhibited by *AnnAt8* overexpressing tobacco transgenic seedlings. Co-expression of *DREB2A* in *AnnAt4* overexpression line also showed an upregulation of *DREB2A* in normal condition, while in NaCl stress it showed a down regulation which could be the reason of drought sensitive phenotype of 35S:AnnAt4 transgenic plants [14]. Possible reasons for the different behaviors of AnnAt4 and AnnAt8 could be the different regulatory mechanisms involved under stress condition. NCED3 is the rate-limiting enzyme of ABA biosynthesis pathway. The higher *NtNCED3* transcript level in *AnnAt8* transgenic seedlings possibly increased the ABA level, inducing ABA-dependent abiotic stress defense pathways. However, AnnAt4 responses shown in drought stress was different where *AtNCED3* showed an upregulation under normal conditions, while it showed a down regulation on drought treatment [14]. This different gene regulatory mechanism needs further investigation.

Osmolytes like proline and sucrose help in maintaining cytosolic osmotic potential during stress conditions [59]. An increase in the *NtP5CS* transcript level, which was consistent with enhanced proline content of *AnnAt8* transgenic lines under normal and stress condition, suggests a possibility of AnnAt8 mediated modulation of *NtP5CS* expression. Previous expression studies made on *AnnAt4* overexpression line in *Arabidopsis* showed a down regulation of *P5CS1* on NaCl treatment [14] and conferred a stress-sensitive phenotype. Sucrose synthase (SUSY) is a key enzyme of the sucrose synthesis pathway. It has been reported that different abiotic stress conditions induce the expression of SUSY [60,61]. Consistent with these reports, the relatively higher expression of *NtSUSY* in the *AnnAt8* transgenic seedlings in comparison to WT under salt stress, also favors stress tolerant phenotype of tobacco transgenic lines.

ERF is a transcription factor which binds to GCC box in the promoter region of stress-inducible genes. Previous reports claim that it responds specifically to pathogen-induced ethylene-mediated signaling. However, recent findings suggest that there is crosstalk between the ERF mediated responses [62]. NtERF5 is a transcription factor, which works in ethylene-dependent and independent manner during different abiotic stress (high salinity, cold, and drought) conditions [63]. Our findings also showed its upregulation in the *AnnAt8* transgenic tobacco plants. Earlier overexpression studies of *ERF* genes support its role in biotic and abiotic stress tolerance [58,64,65].

Expression of NtERD10C and NtERD10D generally increases under osmotic stress. We observed a significantly higher expression of *NtERD10C* and *NtERD10D* in the *AnnAt8* transgenic tobacco plants compared to WT under salt stress treatment. ERD10C and ERD10D belong to LEA family of proteins, which are well known for their role in water holding capacity, and they protect the labile enzymes and the macromolecular structure of the cells, thus help during the dehydration stress. The upregulation of these genes in the *AnnAt8* transgenic plants under NaCl stress condition probably led to better adaptation in early stages of dehydration stress. We also observed a higher expression of *NtMnSOD*, *NtCAT* and *NtAPX* in the transgenic seedlings in comparison to the WT under salt stress condition. Higher accumulation of the transcripts of these genes during stress condition with marginal changes in transcript level under normal growth condition indicates that there might be some possibility of post-translational changes in the AnnAt8 protein, which may enhance its activity under stress condition [20].

SAMDC is a key enzyme of the polyamine biosynthesis pathway [66]. In different stress conditions like drought, salinity and cold, polyamine synthesis is developed [67]. *SAMDC* gene expression is regulated through ABRE and other stress responsive transcription factors [68]. Increased levels of *NtNCED3* in seedling stage in the present analysis correlates well with the increased expression of *SAMDC* gene [69,70]. Another important salt stress inducible gene is *SOS1*, which is regulated by Ca^2+^ mediated signaling. Its antiporter activity is regulated by SOS2 and SOS3, which are upstream components of the SOS signaling; SOS2 is a kinase and SOS3 is a Ca^2+^ binding protein [71]. Upregulation of *SOS1* in *AnnAt8* transgenic lines was observed under salt stress. Previous report on the coexpression of *AtSOS1* in *Atann1* *Arabidopsis* root showed a down regulation in relation to WT under salt stress and it was concluded that the down regulation of *AtSOS1* in a*nnAt1* mutant possibly contributed to poor germination of the mutant under saline condition [21,51]. An increased seed germination rate observed in *AnnAt8* transgenic under salt stress suggests the possibility of AnnAt8 mediated regulation of *NtSOS1*, which may possibly minimize the Na^+^ toxicity effect on seed germination.

## 5. Conclusions

In conclusion, the *AnnAt8* overexpressing tobacco and *Arabidopsis* transgenic plants showed enhanced tolerance to some abiotic stresses. Seed germination under stress is an important phenomenon, which affects the plant stand in stressed soils after germination, which ultimately leads to varied productivity. The transgenic plants showed enhanced seed germination and seedling growth, particularly under salt and dehydration stresses. AnnAt8 expression also mitigated oxidative stress condition. The enhanced stress tolerance is possibly associated with the upregulation of some stress-regulated genes in *AnnAt8* transgenic lines under stress treatments. The expression of these genes has been shown to be involved in stress tolerances in previous studies. The gene-regulatory response of AnnAt8 appears to be similar to AnnAt1 but looks different from AnnAt4 with respect to salt and drought tolerant properties (as reported in earlier studies). These different gene regulatory responses exhibited by annexins in *Arabidopsis* needs further investigation for a better understanding of their mechanism of action under normal and stress conditions, which can vary among the *Arabidopsis* annexin family. This study will help understand the functional redundancy and diversity present within the *Arabidopsis* annexin family.

## Supplementary Materials

Supplementary Materials can be found at www.mdpi.com/2223-7747/5/2/18/s1.

## Abbreviations

ABA, Abscisic acid; PEG, Polyethylene glycol; *AnnAt8,* Arabidopsis annexin-8; APX , Ascorbate peroxidase; CAT, Catalase; MV, Methyl Viologen; PSII, photosystem II; SOD, Superoxide dismutase.

## References

[1] Wang W., Vinocur B., Altman A. (2003). Plant responses to drought, salinity and extreme temperatures: Towards genetic engineering for stress tolerance. Planta.

[2] Apel K., Hirt H. (2004). Reactive oxygen species: Metabolism, oxidative stress, and signal transduction. Annu. Rev. Plant Biol..

[3] Hasegawa P.M. (2013). Sodium (Na^+^) homeostasis and salt tolerance of plants. Environ. Exp. Bot..

[4] Hirayama T., Shinozaki K. (2010). Research on plant abiotic stress responses in the post-genome era: Past, present and future. Plant J..

[5] Shavrukov Y. (2013). Salt stress or salt shock: Which genes are we studying?. J. Exp. Bot..

[6] Claeys H., Van Landeghem S., Dubois M., Maleux K., Inzé D. (2014). What is stress? Dose-response effects in commonly used *in vitro* stress assays. Plant Physiol..

[7] Moss S., Morgan R. (2004). The annexins. Genome Biol..

[8] Clark G.B., Morgan R.O., Fernandez M.P., Roux S.J. (2012). Evolutionary adaptation of plant annexins has diversified their molecular structures, interactions and functional roles. New Phytol..

[9] Gerke V., Creutz C.E., Moss S.E. (2005). Annexins: Linking Ca^2+^ signalling to membrane dynamics. Nat. Rev. Mol. Cell Biol..

[10] Jami S.K., Clark G.B., Ayele B.T., Ashe P., Kirti P.B. (2012). Genome-wide comparative analysis of annexin superfamily in plants. PLoS ONE.

[11] Davies J. (2014). Annexin-mediated calcium signalling in plants. Plants.

[12] Clark G.B., Sessions A., Eastburn D.J., Roux S.J. (2001). Differential expression of members of the annexin multigene family in *Arabidopsis*. Plant Physiol..

[13] Hofmann A. (2004). Annexins in the plant kingdom: Perspectives and potentials. Annexins.

[14] Huh S.M., Noh E.K., Kim H.G., Jeon B.W., Bae K., Hu H.C., Kwak J.M., Park O.K. (2010). Arabidopsis annexins AnnAt1 and AnnAt4 interact with each other and regulate drought and salt stress responses. Plant Cell Physiol..

[15] Wang X., Ma X., Wang H., Li B., Clark G., Guo Y., Roux S., Sun D., Tang W. (2015). Proteomic study of microsomal proteins reveals a key role for Arabidopsis annexin 1 in mediating heat stress-induced increase in intracellular calcium levels. Mol. Cell. Proteom..

[16] Zhu J., Yuan S., Wei G., Qian D., Wu X., Jia H., Gui M., Liu W., An L., Xiang Y. (2014). Annexin5 is essential for pollen development in arabidopsis. Mol. Plant.

[17] Zhu J., Wu X., Yuan S., Qian D., Nan Q., An L., Xiang Y. (2014). Annexin5 plays a vital role in *Arabidopsis* pollen development via Ca^2+^-dependent membrane trafficking. PLoS ONE.

[18] Jia F., Wang C., Huang J., Yang G., Wu C., Zheng C. (2015). SCF E3 ligase PP2-B11 plays a positive role in response to salt stress in *Arabidopsis*. J. Exp. Bot..

[19] Mortimer J.C., Laohavisit A., Macpherson N., Webb A., Brownlee C., Battey N.H., Davies J.M. (2008). Annexins: Multifunctional components of growth and adaptation. J. Exp. Bot..

[20] Konopka-Postupolska D., Clark G., Goch G., Debski J., Floras K., Cantero A., Fijolek B., Roux S., Hennig J. (2009). The role of Annexin 1 in drought stress in Arabidopsis. Plant Physiol..

[21] Lee S., Lee E.J., Yang E.J., Lee J.E., Park A.R., Song W.H., Park O.K. (2004). Proteomic identification of annexins, calcium-dependent membrane binding proteins that mediate osmotic stress and abscisic acid signal transduction in Arabidopsis. Plant Cell Online.

[22] Qiao B., Zhang Q., Liu D., Wang H., Yin J., Wang R., He M., Cui M., Shang Z., Wang D. (2015). A calcium-binding protein, rice annexin OsANN1, enhances heat stress tolerance by modulating the production of H_2_O_2_. J. Exp. Bot..

[23] Zhou M.L., Yang X.B., Zhang Q., Zhou M., Zhao E.Z., Tang Y.X., Zhu X.M., Shao J.R., Wu Y.M. (2013). Induction of annexin by heavy metals and jasmonic acid in *Zea*
*mays*. Funct. Integr. Genom..

[24] Laohavisit A., Davies J.M. (2011). Annexins. New Phytol..

[25] Balasubramanian K., Bevers E.M., Willems G.M., Schroit A.J. (2001). Binding of annexin v to membrane products of lipid peroxidation. Biochemistry.

[26] Laohavisit A., Mortimer J.C., Demidchik V., Coxon K.M., Stancombe M.A., Macpherson N., Brownlee C., Hofmann A., Webb A.A.R., Miedema H. (2009). *Zea mays* Annexins modulate cytosolic free Ca^2+^ and generate a Ca^2+^-permeable conductance. Plant Cell Online.

[27] Laohavisit A., Brown A.T., Cicuta P., Davies J.M. (2010). Annexins: Components of the calcium and reactive oxygen signaling network. Plant Physiol..

[28] Laohavisit A., Shang Z., Rubio L., Cuin T.A., Véry A.A., Wang A., Mortimer J.C., Macpherson N., Coxon K.M., Battey N.H. (2012). Arabidopsis Annexin1 mediates the radical-activated plasma membrane Ca^2+^- and K^+^-permeable conductance in root cells. Plant Cell Online.

[29] Richards S.L., Laohavisit A., Mortimer J.C., Shabala L., Swarbreck S.M., Shabala S., Davies J.M. (2014). Annexin 1 regulates the H_2_O_2_-induced calcium signature in *Arabidopsis thaliana* roots. Plant J..

[30] Arpat A., Waugh M., Sullivan J.P., Gonzales M., Frisch D., Main D., Wood T., Leslie A., Wing R., Wilkins T. (2004). Functional genomics of cell elongation in developing cotton fibers. Plant Mol. Biol..

[31] Cantero A., Barthakur S., Bushart T., Chou S., Morgan R., Fernandez M., Clark G., Roux S. (2006). Expression profiling of the *Arabidopsis* annexin gene family during germination, de-etiolation and abiotic stress. Plant Physiol. Biochem..

[32] Feng Y.M., Wei X.K., Liao W.X., Huang L.H., Zhang H., Liang S.C., Peng H. (2013). Molecular analysis of the annexin gene family in soybean. Biol. Plant..

[33] He M., Yang X., Cui S., Mu G., Hou M., Chen H., Liu L. (2015). Molecular cloning and characterization of annexin genes in peanut (*Arachis hypogaea* L.). Gene.

[34] Jami S.K., Dalal A., Divya K., Kirti P.B. (2009). Molecular cloning and characterization of five annexin genes from Indian mustard (*Brassica juncea* l. Czern and coss). Plant Physiol. Biochem..

[35] Lu Y., Ouyang B., Zhang J., Wang T., Lu C., Han Q., Zhao S., Ye Z., Li H. (2012). Genomic organization, phylogenetic comparison and expression profiles of annexin gene family in tomato (*Solanum lycopersicum*). Gene.

[36] Yadav D., Ahmed I., Kirti P.B. (2015). Genome-wide identification and expression profiling of annexins in brassica rapa and their phylogenetic sequence comparison with *B. juncea* and *A. thaliana* annexins. Plant Gene.

[37] Yan H., Luo Y., Jiang Z., Wang F., Zhou B., Xu Q. (2016). Cloning and expression characterization of four annexin genes during germination and abiotic stress in *Brassica rapa* subsp. Rapa “tsuda”. Plant Mol. Biol. Report..

[38] Ji W., Koh J., Li S., Zhu N., Dufresne C.P., Zhao X., Chen S., Li J. (2015). Quantitative proteomics reveals an important role of gscbrlk in salt stress response of soybean. Plant Soil.

[39] Zhang H., Han B., Wang T., Chen S., Li H., Zhang Y., Dai S. (2012). Mechanisms of plant salt response: Insights from proteomics. J. Proteome Res..

[40] Chu P., Chen H., Zhou Y., Li Y., Ding Y., Jiang L., Tsang E.W.T., Wu K., Huang S. (2011). Proteomic and functional analyses of *Nelumbo nucifera* annexins involved in seed thermotolerance and germination vigor. Planta.

[41] Zhang Y., Xu L., Zhu X., Gong Y., Xiang F., Sun X., Liu L. (2012). Proteomic analysis of heat stress response in leaves of radish (*Raphanus sativus* L.). Plant Mol. Biol. Report..

[42] Steffen J.G., Kang I.H., Macfarlane J., Drews G.N. (2007). Identification of genes expressed in the *Arabidopsis* female gametophyte. Plant J..

[43] Wuest S.E., Vijverberg K., Schmidt A., Weiss M., Gheyselinck J., Lohr M., Wellmer F., Rahnenführer J., von Mering C., Grossniklaus U. (2010). Arabidopsis female gametophyte gene expression map reveals similarities between plant and animal gametes. Curr. Biol..

[44] Clough S.J., Bent A.F. (1998). Floral dip: A simplified method for *Agrobacterium*-mediated transformation of *Arabidopsis thaliana*. Plant J..

[45] Horsch R.B., Fry J.E., Hoffmann N.L., Eichholtz D., Rogers S.G., Fraley R.T. (1985). A simple and general method for transferring genes into plants. Science.

[46] Hiscox J.D., Israelstam G.F. (1979). A method for the extraction of chlorophyll from leaf tissue without maceration. Can. J. Bot..

[47] Heath R.L., Packer L. (1968). Photoperoxidation in isolated chloroplasts: I. Kinetics and stoichiometry of fatty acid peroxidation. Arch. Biochem. Biophys..

[48] Bates L., Waldren R., Teare I. (1973). Rapid determination of free proline for water-stress studies. Plant Soil.

[49] Livak K.J., Schmittgen T.D. (2001). Analysis of relative gene expression data using real-time quantitative pcr and the 2^−ΔΔct^ method. Methods.

[50] Kumar K., Kirti P. (2011). Differential gene expression in *Arachis diogoi* upon interaction with peanut late leaf spot pathogen, *Phaeoisariopsis personata* and characterization of a pathogen induced cyclophilin. Plant Mol. Biol..

[51] Laohavisit A., Richards S.L., Shabala L., Chen C., Colaço R.D.D.R., Swarbreck S.M., Shaw E., Dark A., Shabala S., Shang Z. (2013). Salinity-induced calcium signaling and root adaptation in Arabidopsis require the calcium regulatory protein Annexin1. Plant Physiol..

[52] Faize M., Burgos L., Faize L., Piqueras A., Nicolas E., Barba-Espin G., Clemente-Moreno M., Alcobendas R., Artlip T., Hernandez J. (2011). Involvement of cytosolic ascorbate peroxidase and Cu/Zn-superoxide dismutase for improved tolerance against drought stress. J. Exp. Bot..

[53] Dalal A., Kumar A., Yadav D., Gudla T., Viehhauser A., Dietz K.J., Kirti P.B. (2014). Alleviation of methyl viologen-mediated oxidative stress by *Brassica juncea* annexin-3 in transgenic *Arabidopsis*. Plant Sci..

[54] Clark G.B., Rafati D.S., Bolton R.J., Dauwalder M., Roux S.J. (2000). Redistribution of annexin in gravistimulated pea plumules. Plant Physiol. Biochem..

[55] Clark G.B., Dauwalder M., Roux S.J. (1998). Immunological and biochemical evidence for nuclear localization of annexin in peas. Plant Physiol. Biochem..

[56] Kovács I., Ayaydin F., Oberschall A., Ipacs I., Bottka S., Pongor S., Dudits D., Tóth É.C. (1998). Immunolocalization of a novel annexin-like protein encoded by a stress and abscisic acid responsive gene in alfalfa. Plant J..

[57] Zhu J.K. (2002). Salt and drought stress signal transduction in plants. Annu. Rev. Plant Biol..

[58] Umezawa T., Fujita M., Fujita Y., Yamaguchi-Shinozaki K., Shinozaki K. (2006). Engineering drought tolerance in plants: Discovering and tailoring genes to unlock the future. Curr. Opin. Biotechnol..

[59] Sharma S., Villamor J.G., Verslues P.E. (2011). Essential role of tissue-specific proline synthesis and catabolism in growth and redox balance at low water potential. Plant Physiol..

[60] Dejardin A., Sokolov L., Kleczkowski L. (1999). Sugar/osmoticum levels modulate differential abscisic acid-independent expression of two stress-responsive sucrose synthase genes in Arabidopsis. Biochem. J..

[61] An X., Chen Z., Wang J., Ye M., Ji L., Wang J., Liao W., Ma H. (2014). Identification and characterization of the *Populus* sucrose synthase gene family. Gene.

[62] Park J.M., Park C.J., Lee S.B., Ham B.K., Shin R., Paek K.H. (2001). Overexpression of the tobacco tsi1 gene encoding an EREBP/AP2-type transcription factor enhances resistance against pathogen attack and osmotic stress in tobacco. Plant Cell.

[63] Zhai Y., Wang Y., Li Y., Lei T., Yan F., Su L., Li X., Zhao Y., Sun X., Li J. (2013). Isolation and molecular characterization of *Gmerf7*, a soybean ethylene-response factor that increases salt stress tolerance in tobacco. Gene.

[64] Medina J., Catalá R., Salinas J. (2011). The CBFs: Three *Arabidopsis* transcription factors to cold acclimate. Plant Sci..

[65] Robert-Seilaniantz A., Grant M., Jones J.D. (2011). Hormone crosstalk in plant disease and defense: More than just jasmonate-salicylate antagonism. Annu. Rev. Phytopathol..

[66] Rangan P., Subramani R., Kumar R., Singh A.K., Singh R. (2014). Recent advances in polyamine metabolism and abiotic stress tolerance. BioMed. Res. Int..

[67] Groppa M., Benavides M. (2008). Polyamines and abiotic stress: Recent advances. Amino Acids.

[68] Basu S., Roychoudhury A., Sengupta D. (2014). Deciphering the role of various cis-acting regulatory elements in controlling samdc gene expression in rice. Plant Signal. Behav..

[69] Bitrián M., Zarza X., Altabella T., Tiburcio A.F., Alcázar R. (2012). Polyamines under abiotic stress: Metabolic crossroads and hormonal crosstalks in plants. Metabolites.

[70] Urano K., Yoshiba Y., Nanjo T., Igarashi Y., Seki M., Sekiguchi F., Yamaguchi-Shinozaki K., Shinozaki K. (2003). Characterization of *Arabidopsis* genes involved in biosynthesis of polyamines in abiotic stress responses and developmental stages. Plant Cell Environ..

[71] Isayenkov S. (2012). Physiological and molecular aspects of salt stress in plants. Cytol. Genet..

