# Supplementary material for: Overexpression of Arabidopsis AnnAt8 Alleviates Abiotic Stress in Transgenic Arabidopsis and Tobacco
Source: Plants (Basel). 2016 Apr 14;5(2):18. doi: 10.3390/plants5020018 (PMC4931398; doi:10.3390/plants5020018)
Supplement: Supplementary file 1 [file plants-05-00018-s001.pdf]

Supplementary Materials: Overexpression of *Arabidopsis AnnAt8* Alleviates Abiotic Stress in Transgenic *Arabidopsis* and Tobacco

Deepanker Yadav, Israr Ahmed, Pawan Shukla, Prasanna Boyidi and Pulugurtha Bharadwaja Kirti

Table S1. Primers used in the amplification of *AnnAt8* for the construction of recombinant vectors.

Cloning	Primer Name	Primer Sequence
pRT100	*AnnAt8* (ORF1) *Nco*I F	5'-CCCATGGCCACCATTGTTTCTCC-3'
	AnnAt8 (ORF1) *Xba*I R	5'-CTCTAGACTAAAGTTGTTTTCCATGTCCT-3'
pEGAD	*AnnAt8*(ORF2) *Eco*RI F	5'-AAGAATTCATGGCCACCATTGTTTCTCC-3'
	AnnAt8(ORF2) *Sma*I R	5'-AACCCGGGCTAAAGTTGTTTTCCATGTCCT-3'

Table S2. Primers used in the amplification of *AnnAt8* and *NptII* for the confirmation of the transgene presence in the putative transformants.

Gene	Primer Sequence
AnnAt8	F-5'-CCCATGGCCACCATTGTTTCTCC-3'
	R-5'-CTCTAGACTAAAGTTGTTTTCCATGTCCT-3'
NptII	F-5'-GAGGCTATTCGGCTATGACTG-3'
	R-5'-ATCGGGAGCGGCGATACCGTA-3'

Table S3. Primers used for semi quantitative and real time PCR analysis of various genes expressed under stress.

Gene	Gene Bank ID	Primer Sequence
NtDREB3	EU727157.1	F-5'-ATGGCTTGGCACTTTCCCTT-3'
		R-5'-ATATTCTTGGCGTCGGAGGA-3'
NtSAMDC	U91924.1	F-5'-CAGTCGTTTCCTCACCGTCA-3'
		R-5'-ATAGGTCCTGCAGAGGCAGA-3'
NtAPX	U15933.1	F-5'-GTTTGGGCTTTTCTCCTCGAC-3'
		R-5'-GGAGCATAAGAGGAGCGCAA-3'
NtMnSOD	AB093097.1	F-5'-TCCCCTACGACTATGGAGCA-3'
		R-5'-CGGTATGCAATTTGGCGACG-3'
NtERF5	AY655738.1	F-5'-GGATTGTCTCCTGCTGCTGT-3'
		R-5'-GCTCTTCTAATAACTCAGCACCC-3'
NtERD10D	AB049338.1	F-5'-GCACGAGGGAAGAAGAGAAGG-3'
		R-5'-TGGAGGCGCCACTTCCTC-3'
NtERD10C	AB049337.1	F-5'-AAAGCCAACTCATGCCCAAG-3'
		R-5'-AGAGCTGCTACTTGATCGATGG-3'
NtNCED3	JX101472.1	F-5'-TGTCTGAAATGATCCGGGGC-3'
		R-5'-AGTTTCCGGCTCTTCCCAAG-3'
NtSUSY	AB055497.1	F-5'-CACGGATATTTCGCCCAGGA-3'
		R-5'-GCAGCAGCCGAGTAGCAATA-3'
NtP5CS	HM854026.1	F-5'-GCTGCTCAACAGGCTGGATA-3'
		R-5'-CCATCAGCAACCTCCGTTCT-3'
NtSOS1	XM_009789739.1	F-5'-CAAATGTTATCCCCCGAAAGC-3'
		R-5'-CGGAGAACCTGAGGAAATGTGA-3'

Table S3. *Cont.*

Gene	Gene Bank ID	Primer Sequence
NtActin	EU938079.1	F-5'-TTTCCGATGCCCTGAAGTCC-3'<br>R-5'-CATAGTCGAACCGCCACTGA-3'
NtCAT	U93244.1	F-5'-GGCCGCTACAACTCTCTCTTT-3'<br>R-5'-ACAGGACCTCTTGCACCAAC-3'
Nt18S	AJ236016.1	F-5'-CCAGGTCCAGACATAGTAAG-3'<br>R-5'-GTACAAAGGGCAGGGACGTA-3'

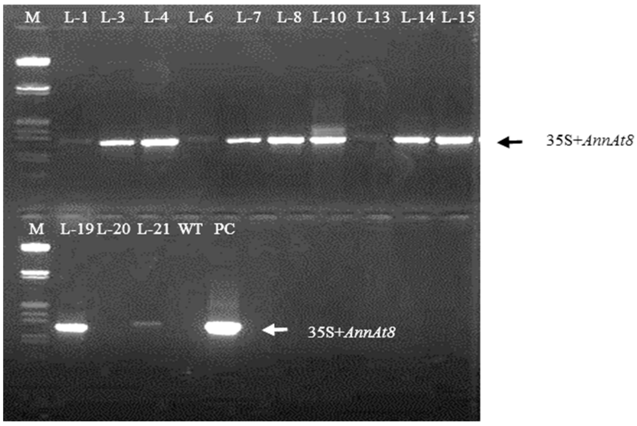

Figure S1. Confirmation of putative transgenic lines by PCR using *Arabidopsis* genomic DNA. A representative PCR gel picture showing amplified product for 35S+AnnAt8 from thirteen putative *AnnAt8* transgenic (T_1) *Arabidopsis* plants. Letter 'PC' corresponds to positive control for the PCR using plasmid (pCAMBIA2300::*AnnAt8*) as a template. Letter 'WT' corresponds to negative control for the PCR using DNA from untransformed plant. Letter 'M' correspond to λ *Hind*III/*Eco*RI DNA Marker.

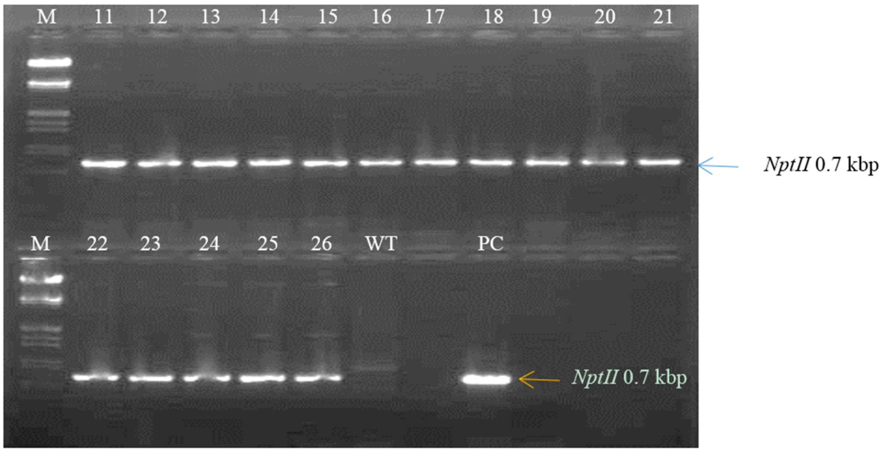

Figure S2. A representative PCR gel picture showing 0.7 Kb amplified product for *NptII* from sixteen putative *AnnAt8* transgenic (T_0) tobacco plants. Letter 'PC' corresponds to positive control for the PCR using plasmid (pCAMBIA2300::*AnnAt8*) as a template. Letter 'WT' corresponds to negative control for the PCR using DNA from untransformed plant. Letter 'M' correspond to λ *Hind*III/*Eco*RI DNA Marker.
